# Identification of CTLA2A, DEFB29, WFDC15B, SERPINA1F and MUP19 as Novel Tissue-Specific Secretory Factors in Mouse

**DOI:** 10.1371/journal.pone.0124962

**Published:** 2015-05-06

**Authors:** Jibin Zhang, Jinsoo Ahn, Yeunsu Suh, Seongsoo Hwang, Michael E. Davis, Kichoon Lee

**Affiliations:** 1 Department of Animal Sciences, The Ohio State University, Columbus, Ohio, United States of America; 2 The Ohio State University Interdisciplinary Ph.D. Program in Nutrition, The Ohio State University, Columbus, Ohio, United States of America; 3 Animal Biotechnology Division, National Institute of Animal Science, RDA, Gyeonggi, Republic of Korea; East Tennessee State University, UNITED STATES

## Abstract

Secretory factors in animals play an important role in communication between different cells, tissues and organs. Especially, the secretory factors with specific expression in one tissue may reflect important functions and unique status of that tissue in an organism. In this study, we identified potential tissue-specific secretory factors in the fat, muscle, heart, lung, kidney and liver in the mouse by analyzing microarray data from NCBI’s Gene Expression Omnibus (GEO) public repository and searching and predicting their subcellular location in GeneCards and WoLF PSORT, and then confirmed tissue-specific expression of the genes using semi-quantitative PCR reactions. With this approach, we confirmed 11 lung, 7 liver, 2 heart, 1 heart and muscle, 7 kidney and 2 adipose and liver-specific secretory factors. Among these genes, 1 lung-specific gene - CTLA2A (cytotoxic T lymphocyte-associated protein 2 alpha), 3 kidney-specific genes - SERPINA1F (serpin peptidase inhibitor, Clade A, member 1F), WFDC15B (WAP four-disulfide core domain 15B) and DEFB29 (defensin beta 29) and 1 liver-specific gene - MUP19 (major urinary protein 19) have not been reported as secretory factors. These genes were tagged with hemagglutinin at the 3’end and then transiently transfected to HEK293 cells. Through protein detection in cell lysate and media using Western blotting, we verified secretion of the 5 genes and predicted the potential pathways in which they may participate in the specific tissue through data analysis of GEO profiles. In addition, alternative splicing was detected in transcripts of CTLA2A and SERPINA1F and the corresponding proteins were found not to be secreted in cell culture media. Identification of novel secretory factors through the current study provides a new platform to explore novel secretory factors and a general direction for further study of these genes in the future.

## Introduction

The secretory factors are a large group of proteins synthesized by ribosomes bound to rough endoplasmic reticulum (ER). During the process of protein synthesis, the proteins are directed from the cytosolic face of the ER membrane to the ER lumen by the ER signal sequences at the N-terminus of the proteins [1]. The proteins in the ER lumen are subsequently packaged into transport vesicles that fuse with the cis-Golgi vesicles. Cis-Golgi vesicles then move toward the plasma membrane and change to trans-Golgi cisternae [2]. Some secretory factors such as hormones are stored in secretory vesicles and are only released upon triggers of hormonal or neural signals. Other secretory factors such as those found in the extracellular matrix are continuously secreted and exist in all cell types [3].

Due to the ubiquitous property of secretory factors, they play an important role in various cells, organs and systems in animals. As increasing numbers of secretory factors are explored, functions of one cell type and tissue and connections between different cell types and tissues become further understood and complex networks in living organisms become extensively revealed. For example, fat tissue was once regarded only as a lipid reservoir for excess energy. However, since the discovery of leptin by Zhang et al. [4], perceptions about fat tissue have gradually changed. Leptin is a protein secreted by mature adipocytes, and can regulate food intake and body fat mass by binding to its receptor in the hypothalamus. Since the identification of leptin, more than 100 secretory factors have been identified that are produced and released by adipose tissue [5]. Among these factors, some adipose-specific secretory proteins such as adiponectin, resistin and visfatin are involved in the immune system [6], while some proteins such as angiotensinogen [7] and plasminogen activator inhibitor type I [8] are related to vascular function. Therefore, adipose tissue is now thought of, not only as a lipid-storing organ, but also as an endocrine organ that maintains intensive cross talk with other organs.

Since tissue-specific expression of one novel secretory factor usually indicates novel function of that tissue, it is necessary to explore novel tissue-specific secretory factors. Tissue specific expression of various genes can be detected by microarrays with measurements of transcript abundance in various tissues. There are thousands of microarray data records from various studies with open access in the Gene Expression Omnibus (GEO) database on the NCBI website, providing a useful tool to explore tissue-specific genes and predict their functions [9]. In our previous study we successfully selected some novel tissue-specific genes in the human and mouse and predicted their potential function by taking advantage of this database [10]. The subcellular location of various proteins can also be found in some databases such as GeneCards [11] and UniProt [12] or predicted by WoLF PSORT [13]. These powerful bioinformatic tools greatly facilitate the selection of novel secretory factors with tissue specific expression.

The objective of this study is to identify and evaluate novel tissue-specific secretory genes in the fat, muscle, heart, lung, liver and kidney in the mouse by performing microarray data analysis, literature search, protein location information search and prediction, semi-quantitative PCR analysis and Western blotting after transfection of expression vectors in cell culture. With the bioinformatics approach developed by Song et al. [10], we detected novel tissue-specific genes in the 6 tissues and filtered potential secretory genes by searching and predicting protein location. After confirmation of tissue-specific expression through semi-quantitative PCR analysis, 30 genes were identified in 6 tissues in adult mice. Among them, 11 genes are lung-specific; 7 genes are liver-specific; 2 genes are heart-specific; 1 gene is muscle and heart-specific; 7 genes are kidney-specific and 2 genes are adipose and liver-specific. After the literature study, 5 novel tissue-specific secretory genes were discovered: CTLA2A (cytotoxic T lymphocyte-associated protein 2 alpha) in the lung, SERPINA1F (serpin peptidase inhibitor, Clade A, member 1F), WFDC15B (WAP four-disulfide core domain 15B), DEFB29 (defensin beta 29) in the kidney, and MUP19 (major urinary protein 19) in the liver tissue. The secretory properties of these proteins were detected by Western blot analysis using cell lysate and medium after transient transfection of expression vectors to HEK293 cells. The biological activities in which these genes may participate in the specific tissues were predicted according to GEO profiles under different physiological and pathological conditions. This study provides a novel strategy combining bioinformatics tools and molecular technologies for exploration of novel tissue-specific secretory genes.

## Materials and Methods

### Experimental Animals

Mice raised in a mouse housing facility at The Ohio State University with free access to feed were euthanized by CO_2_ inhalation and subsequent cervical dislocation at 3 months of age. Then white adipose tissue, muscle, heart, lung, liver and kidney were harvested (n = 3). All procedures were approved by the Institutional Animal Care and Use Committee (IACUC) of The Ohio State University. After tissue collection, all of the tissues were snap-frozen immediately in liquid nitrogen and stored at -80°C for total RNA isolation.

### Data Mining and Literature Search

In order to find the secretory factors that are highly expressed in the adipose tissue, muscle, heart, lung, liver and kidney in the adult mouse, the microarray expression records in GDS3142, which is one Gene Expression Omnibus DataSet (GDS) available on the NCBI web site, were analyzed following the methods proposed by Song et al. [10]. In brief, expression records for each gene in the 6 different tissues were derived and ratios were calculated between expression in each tissue and average expression in the other 5 tissues; so there was one list of ratios for each tissue. After ranking the genes in descending order according to each list of ratios, the top 200–300 genes were selected for each tissue. For genes with several spots on the microarray, only one record was kept. Then, each of these genes was checked in Genecards (http://www.genecards.org/) and UniProt (http://www.uniprot.org/) and predicted by WoLF PSORT (http://www.genscript.com/psort/wolf_psort.html) to see whether the corresponding protein was secreted or not. Finally, only the secretory genes were kept and a literature search was conducted in Pubmed (http://www.ncbi.nlm.nih.gov/pubmed) and Google Scholar (http://scholar.google.com/) to check whether there are reports concerning high expression of each gene and secretion of the protein in certain tissues. After the 5 novel secretory factors were selected, their functions in specific tissues were further predicted based on analysis of various GEO profiles. Function of CTLA2A was predicted based on GDS3950, GDS4582, GDS4914 and GDS2709; function of MUP19 was predicted based on GDS1261, GDS1053, GDS279, GDS1517 and GDS1374; function of SERPINA1F was predicted based on GDS2031, GDS4316 and GDS3675; function of WFDC15B was predicted based on GDS2030, GDS4316, GDS3675, GDS2817, GDS1583 and GDS4449; and function of DEFB29 was predicted based on GDS2031, GDS4316, GDS1583, GDS4839 and GDS3612.

### cDNA Synthesis and Semi-quantitative PCR Analysis

For the genes whose tissue-specific expressions are not reported in existing publications, we conducted a semi-quantitative PCR to detect their expression in different tissues. Total RNA was isolated from the adipose tissue, muscle, heart, lung, liver and kidney of the adult mice using Trizol reagent (Invitrogen, Carlsbad, CA, USA) and then reverse-transcribed to cDNA using moloney murine leukemia virus (M-MLV) reverse transcriptase (Invitrogen) and oligo dT. The conditions for reverse transcription were 65°C for 5 min, 37°C for 50 min and 70°C for 15 min. After the RNA was reverse-transcribed to cDNA, PCR was conducted with 1μL of cDNA, 0.2 μL of 10mM deoxynucleotide triphosphate mix (dNTP), 1 μL of 10×ThermopoIII (Mg-free) reaction buffer, 0.2 μL of 100 mM MgSO_4_, 0.2 nM of each of the forward and reverse primers, 0.05 μL of Taq DNA polymerase (New England BioLabs, Ipswich, MA, USA) and nuclease-free water up to 10 μL. The cycling parameters for PCR were 95°C for 1 min, followed by 32 to 40 cycles of 94°C for 30 s, 58°C for 30 s and 72°C for 40 s with a final elongation for 10 min. To ensure that equal amounts of cDNA were added to the PCR reaction for different tissues, expression of the cyclophilin (CYC) gene was used as a control and cDNA was diluted until the PCR products of CYC showed similar brightness for different tissues in agarose gel electrophoresis. After the equalization of cDNA, PCR was conducted for the other genes. The PCR primers for all of the genes are listed in S1 Table.

### Vector Construction and Cell Transfection

The subcellular locations of 5 of the selected genes were not found in the literature. To verify secretion of their encoding proteins from cells, we constructed plasmids containing target genes to transfect cells to detect the location of expressed protein. First, coding sequences of these genes were amplified with primers shown in S2 Table. For CTLA2A, 2 forward primers were used to amplify 2 transcripts with alternative start codons. Hemagglutinin (HA) tag sequence, which is underlined in the table, was linked to the 5’ end of all of the reverse primers to add the HA-tag to the C terminal of the target proteins. After gel extraction of the PCR product, the amplified sequences were ligated to the pCR2.1 vector (Invitrogen) using T4 DNA ligase (New England BioLabs). Then, the plasmid with an insertion of each PCR product was transformed to *E*.*coli* cells, which were then spread over plates containing 5-bromo-4-chloro-3-indolyl-beta-D-galacto-pyranoside (X-gal). Because the lacZ gene in the plasmid is disrupted by inserted sequences, the colonies were white, whereas, the plasmids with no insertion had intact lacZ genes expressing β-galactosidase, and its reaction with X-gal stained the colony in a blue color. After selection of positive colonies and incubation in Luria-Bertani (LB) broth, plasmids were extracted using a miniprep Kit (QIAGEN, Valencia, CA, USA) and the direction of inserted genes was verified through restriction enzyme digestion and PCR with M13 primers and the primers of inserted genes. The plasmids with correct orientation of insertion were then digested with HindIII and XhoI (New England BioLabs) and ligated to a pcDNA 3.1 vector, which was also digested with the same enzymes. The constructed vectors were then transformed to E. coli and insertion was verified by digestion with HindIII and XhoI and subsequent electrophoresis in 0.6% agarose gels. After confirmation of the correct ligation, the constructed vectors were extracted using a plasmid midi kit (QIAGEN).

### Transient Transfection of Plasmids in HEK 293 Cells

One day before transfection, human embryonic kidney 293 (HEK293) cells were centrifuged and resuspended in Dulbecco’s modified Eagle’s medium (DMEM, Gibco, Grand Island, NY) supplemented with 10% fetal bovine serum (FBS, Gibco). On the day of transfection, the cells were transfected with pcDNA3.1 vector containing HA-tagged putative secretory proteins or the same vector without an insert using lipofectamine 2000 (Invitrogen) according to the manufacturer’s protocol. The medium was changed to fresh complete growth medium after 6 h. Then, the cells were incubated for 48 h prior to protein extraction for Western blot analysis.

### Western Blot Analysis

After collecting the cell medium, proteins expressed in HEK293 cells were extracted using ice-cold 1× lysis buffer (125 mM Tris-HCl pH 6.8, 0.5% SDS). Proteins in the medium were precipitated using Tricholoroacetic acid (TCA). In brief, the medium was spun down at 1,200 × g to pellet the cell debris and the supernatant was centrifuged at 16,000 × g after adding TCA (25% v/v). The protein pellet was washed 2 times with ice-cold acetone, and then resolved in 2X loading buffer. Equal amounts of proteins were loaded and separated by 10–15% SDS-PAGE and wet-transferred to polyvinylidene difluoride (PVDF) membranes (Bio-Rad, Hercules, CA). After blocking for 30 min in Tris-buffered saline with Tween-20 (TBST) containing 4% nonfat dry milk, the membranes were incubated overnight at 4°C with mouse anti-HA-tag (1:3000; Cell Signaling, Danvers, MA) antibody as a primary antibody. Horseradish peroxidase-linked anti-mouse IgG (1:5000; Cell Signaling) was used as a secondary antibody for an 1-h incubation at room temperature. After detecting signals with ECL plus reagents (GE Healthcare Biosciences, Pittsburgh, PA), proteins were visualized by exposure of the membranes to X-ray films (GE Healthcare Biosciences).

### Statistical Analysis

A mixed model (MIXED) procedure was performed for comparison among more than 2 groups using SAS software (version 9.3, SAS Institute Inc., USA). The DIFF command was used to detect significant differences between pairs of least squares means. Student’s t-test was performed in JMP 10 to compare gene expression between 2 groups. Differences with p-values lower than 0.05 were treated as significant. Statistical values are presented as least squares means with standard errors of the least squares means (SEM).

## Results

### Discovery of Tissue-Specific Secretory Genes

**Fig 1:**
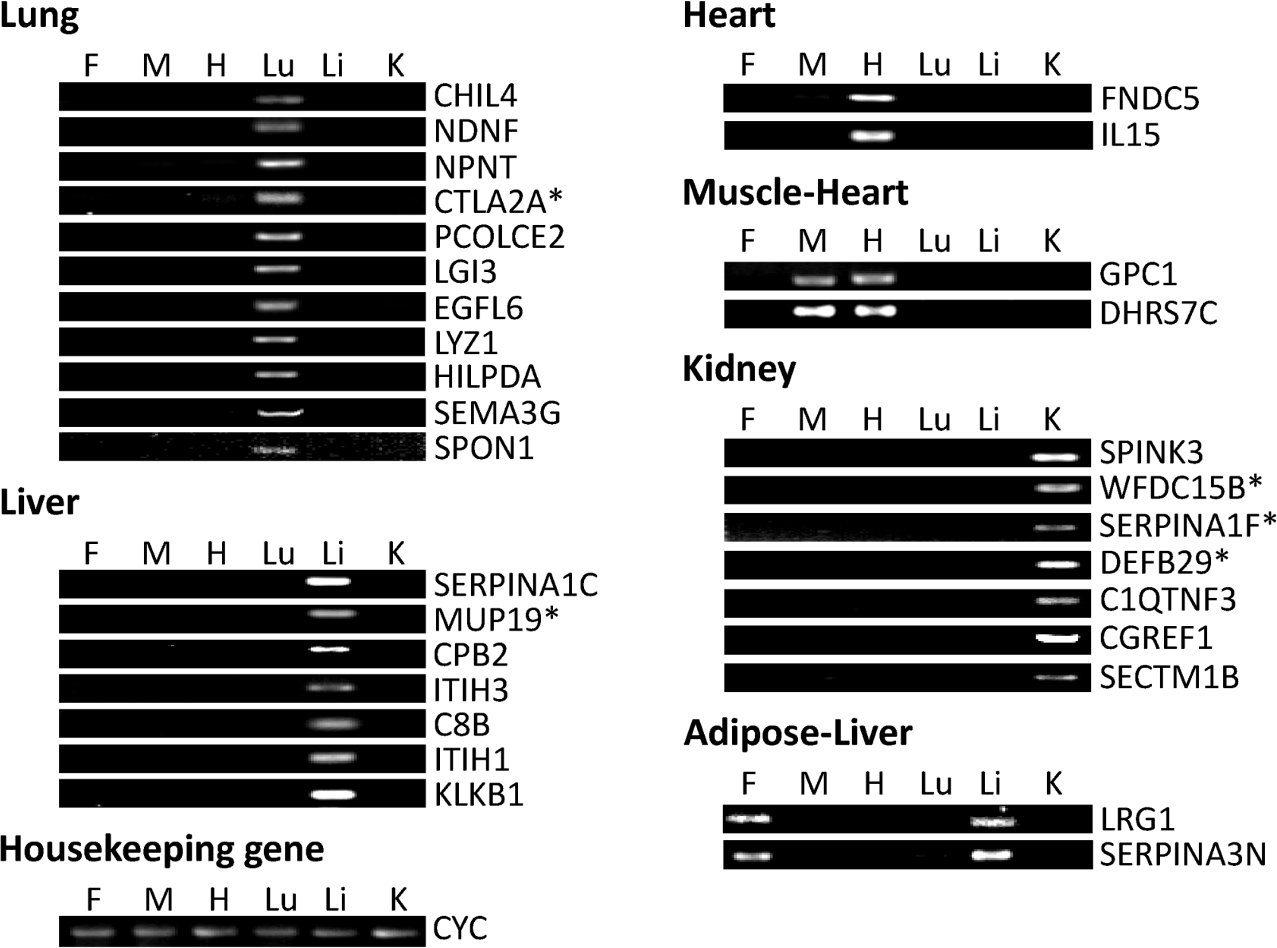
Expression of adult mouse gene transcripts detected by semi-quantitative PCR. Expressions of selected genes in fat (F), muscle (M), heart (H), lung (Lu), liver (Li) and kidney (K) were detected by PCR reaction and 1% agarose gel electrophoresis. Housekeeping gene—cyclophilin (CYC)—was used as a loading control. Genes without reported subcellular locations are marked with asterisks.

Six Excel spreadsheet files were generated with the top 200–300 genes in terms of highest expression in the fat, muscle, heart, lung, liver and kidney in the adult mice based on GDS3142. After conducting searches of the Genecards and Uniprot websites, protein subcellular localization prediction in WoLF PSORT and literature searches in Pubmed and Google Scholar, non-secretory genes and known secretory genes with reported tissue-specific expression consistent with our findings in GDS3142 were deleted and 31 genes were kept with their average expression values in 6 different tissues from GDS3142 listed in Table 1. Among these genes, 11 are more highly expressed in the lung, 7 genes are more highly expressed in the liver; 2 are more highly expressed in the heart, and one—(GPC1) shows nearly equal expression in heart and muscle; one gene (DHRS7C) shows the highest expression in the muscle without much difference in expression in the heart; 7 genes are expressed more highly in the kidney; 2 genes show the highest expression in adipose with little difference in expression in the liver. The expression patterns of these genes were confirmed by semi-quantitative PCR and gel electrophoresis. As shown in Fig 1, both adipose-specific genes showed similar expression in adipose tissue and liver; DHRS7C and GPC1 showed similar expression in muscle and heart; whereas, all other genes only showed detectable expression in one specific tissue.

**Table 1:** Gene expression values of selected genes.

Gene	Adipose	Muscle	Heart	Lung	Liver	Kidney	A/O	Rank	Subcellular Location
CHIL4	103±7^b^ ^c^	93±5^b^ ^c^	102±4^b^ ^c^	**4137±595** ^**a**^	100±2^b^	94±2^c^	42.2	14	Secreted^[^ ^14^ ^]^
NDNF	112±11^c^	83±3^d^	159±11^b^	**1355±83** ^**a**^	75±6^d^ ^e^	72±4^e^	13.5	36	Secreted^[^ ^15^ ^]^
NPNT	78±8^d^	300±22^c^	85±6^d^	**2664±67** ^**a**^	57±1^e^	693±65^b^	11.0	49	EM^[^ ^16^ ^,^ ^17^ ^]^
CTLA2A	550±56^b^	357±15^c^	424±63^b^ ^c^	**3303±228** ^**a**^	127±23^d^	142±13^d^	10.3	52	No report
PCOLCE2	390±47^b^	275±17^c^	279±15^c^	**2146±196** ^**a**^	85±12^d^	88±3^d^	9.6	60	EM^[^ ^18^ ^]^
LGI3	97±5^b^	74±1^c^ ^d^	87±0.3^b^	**584±13** ^**a**^	69±2^d^	77±1^c^	7.2	92	Secreted^[^ ^19^ ^]^
EGFL6	72±2^d^	92±6^c^	69±2^d^ ^e^	**692±66** ^**a**^	61±4^e^	249±66^b^	6.4	130	Secreted^[^ ^20^ ^]^
LYZ1	3867±251^b^	890±72^d^	1780±151^c^	**8801±639** ^**a**^	265±111^e^	184±31^e^	6.3	132	Secreted^[^ ^21^ ^]^
HILPDA	197±17^b^	132±2^c^	178±8^b^	**824±55** ^**a**^	130±7^c^	102±4^d^	5.6	159	Secreted^[^ ^22^ ^]^
SEMA3G	248±23^b^ ^c^	169±8^d^	204±5^c^	**1025±111** ^**a**^	87±4^e^	294±11^b^	5.1	186	Secreted^[^ ^23^ ^]^
SPON1	379±65^b^	190±5^c^	278±7^b^	**1095±85** ^**a**^	90±1^d^	278±39^b^	4.5	237	EM^[^ ^24^ ^]^
SERPINA1C	84±5^d^	109±4^c^	135±12^b^ ^c^	96±22^c^ ^d^	**16792±404** ^**a**^	214±38^b^	143.9	5	Secreted^[^ ^25^ ^]^
MUP19	111±2^b^	844±568^b^ ^c^	118±3^b^	93±4^c^	**22563±315** ^**a**^	96±4^c^	101.0	20	No report
CPB2	60±3^e^	64±3^d^ ^e^	75±3^b^ ^c^	65±3^c^ ^d^ ^e^	**3173±34** ^**a**^	82±7^b^	47.1	67	Secreted^[^ ^26^ ^]^
ITIH3	86±5^b^ ^c^	81±3^c^	91±0.5^b^	85±1^c^	**3774±79** ^**a**^	100±4^b^	34.9	89	Secreted^[^ ^28^ ^,^ ^29^ ^]^
C8B	95±7^c^ ^d^	99±3^b^ ^c^	124±11^b^	92±6^c^ ^d^	**2389±14** ^**a**^	87±3^d^	24.9	121	Secreted^[^ ^30^ ^]^
ITIH1	104±5^b^ ^c^	99±4^c^	111±3^b^	128±15^b^ ^c^	**2003±54** ^**a**^	110±8^b^ ^c^	18.8	154	Secreted^[^ ^28^ ^,^ ^29^ ^]^
KLKB1	82±2^d^	90±3^c^	111±8^b^	88±4^c^ ^d^	**1393±42** ^**a**^	103±3^b^	15.4	176	Secreted^[^ ^31^ ^]^
FNDC5	109±8^c^ ^d^	275±15^b^	**1032±76** ^**a**^	107±1^c^	137±17^c^ ^d^	103±1d	7.1	78	Secreted^[^ ^32^ ^]^
IL15	161±12^c^ ^d^	188±20^c^	**591±83** ^**a**^	122±19^d^ ^e^	86±3^e^	283±6^b^	3.5	246	Secreted^[^ ^33^ ^]^
GPC1	181±5^c^	739±55^b^	**863±2** ^**a**^	155±6^d^	172±2^c^	132±3^e^	3.1	328	EM^[^ ^34^ ^]^
DHRS7C	91±1^c^	**1395±82** ^**a**^	713±32^b^	99±9^c^	92±7^c^	89±6^c^	6.4	171	ER ^[^ ^42^ ^]^
SPINK3	71±4^c^	77±3^b^ ^c^	85±4^b^	73±6^b^ ^c^	79±3^b^ ^c^	**5525±151** ^**a**^	71.6	15	Secreted^[^ ^35^ ^]^
WFDC15B	100±2^c^ ^d^	96±3^d^	135±12^b^	93±5^d^	107±4^c^	**3066±444** ^**a**^	28.9	39	No report
SERPINA1F	77±5^b^ ^c^	70±3^c^	87±2^b^	72±5^c^	77±6^b^ ^c^	**1433±297** ^**a**^	18.7	66	No report
DEFB29	70±4^c^ ^d^	65±1^d^	90±6^b^	74±9^b^ ^c^	75±4^b^ ^c^	**1228±74** ^**a**^	16.4	80	No report
C1QTNF3	73±9^b^ ^c^ ^d^	70±3^b^	62±1^c^	71±1^b^	61±2^d^	**933±59** ^**a**^	13.8	98	Secreted^[^ ^36^ ^]^
CGREF1	86±5^c^	119±8^b^	112±1^b^	89±8^c^	97±10^b^ ^c^	**1027±32** ^**a**^	10.2	132	Secreted^[^ ^37^ ^]^
SECTM1B	86±4^c^	87±4^c^	104±5^b^	88±9^b^ ^c^	98±3^b^	**616±80** ^**a**^	6.7	234	Secreted^[^ ^38^ ^]^
LRG1	**3947±34** ^**a**^	398±11^c^	667±64^b^	544±15^b^	3108±450^**a**^	112±6^d^	4.1	272	Secreted^[^ ^39^ ^,^ ^40^ ^]^
SERPINA3N	**1399±87** ^**a**^	150±20^d^ ^e^	179±4^d^	496±64^c^	1070±175^b^	92±3^e^	3.5	360	Secreted^[^ ^41^ ^]^

^a-e^ Different superscripts within a row indicate significant difference (P<0.05).

The highest expression value for each gene is highlighted in bold font. A/O: ratio between average expression of certain gene in tissue with specific expression of that gene and expression in other tissues. EM: Extracellular matrix; ER: Endoplasmic reticulum.

As shown in Table 1, 25 of the selected genes have been reported to produce extracellular proteins. In the lung-specific genes, CHIL4 encodes Chitinase-like protein 4, which is also called secreted Ym2 protein. It has been identified in respiratory secretions and reported to be increasingly expressed in the allergic lung [14]. Neuron-Derived Neurotrophic Factor (NDNF) was reported to be a secreted glycosylated protein by Kuang et al. [15], but its secretion was reported in the brain and neuron cell culture rather than in the lung. NPNT encodes nephronectin, which is an extracellular matrix protein and has been reported to be secreted from the embryonic kidney [16] and the MC3T3-E1 cell line [17]. PCOLCE2 encodes procollagen C-endopeptidase enhancer 2, a collagen-binding protein that was reported to be secreted in transfected human 293 EBNA-1 cells [18]. Leucine-rich glioma inactivated 3 (LGI3) is a secreted leucine-rich repeat protein. It was reported to be highly expressed in the brain and, thus, has been extensively studied in the nervous system [19], but there is no report in lung. EGFL6 encodes an epidermal growth factor–like protein, which has been reported to be secreted in an osteoblastic-like cell culture [20]. LYZ1 encodes lysozyme, which has been reported to be primarily secreted by serous cells of submucosal glands in the lung [21]. Hypoxia Inducible Lipid Droplet-Associated (HILPDA) was reported to be secreted in COS7 cell culture, but its expression was only reported in fetal kidney and renal cell carcinoma [22]. SEMA3G encodes semaphorin 3G-a member of murine class 3 semaphorin. It is expressed in the lung and kidney and secreted in HEK293-T cells [23]. SPON1 encodes F-spondin, which is an extracellular matrix protein secreted in the floor plate in the embryonic neural tube [24]. Its secretion and function have been extensively studied in the nervous system. However, there are few reports about this gene in the lung.

Among the liver-specific genes, SERPINA1C encodes one isoform of murine a-1-antitrypsin, which is produced in the liver, secreted in serum and circulated to the lung [25]. Secretion of SERPINA1C has also been reported by Paterson and Moore [26]. CPB2 encodes plasma Carboxypeptidase B2, which is secreted from the liver [27]. Both ITIH1 and ITIH3 encode one heavy chain of inter-alpha-trypsin inhibitor (ITI), which is one plasma protease inhibitor, and is synthesized and secreted in a hepatoma HepG2 cell culture [28] and COS7 cell culture [29]. C8B encodes the β subunit of complement component C8, which is a component of the membrane attack complex (MAC) and has been reported to be secreted by hepatocytes into serum. The a, b, and g subunits normally associate with each other before secretion [30]. KLKB1 encodes plasma kallikrein, which is synthesized in the liver [31].

Among heart-specific genes, FNDC5 encodes fibronectin type III domain-containing protein 5, which is a membrane protein proteolytically cleaved and secreted as a hormone peptide—Irisin. Irisin is mainly secreted by cardiac and skeletal muscle, with the former producing more than the latter [32]. IL15 encodes interleukin 15, whose production has been detected in various tissues. This gene produces 2 different isoforms through alternative splicing. The 21-aa signal isoform mRNA is expressed in the heart, thymus, appendix and testis, translated efficiently but not secreted. The 48-aa signal isoform mRNA is expressed in skeletal muscle, placenta, heart, lung, liver, thymus and kidney, translated less efficiently and secreted at a low level [33]. GPC1 encodes glypican-1, which is a cell membrane heparan sulfate proteoglycan. It is attached to the cell membrane by a glycosylphosphatidylinositol (GPI) linkage. The GPI anchor can be cleaved and thus lead to secretion of glypican-1 or shedding into the extracellular matrix [34].

Among the kidney-specific genes, SPINK3 encodes pancreatic secretory trypsin inhibitor in the mouse. It is produced in acinar cells in the pancreas and secreted with digestive enzymes into secretory granules [35]. However, its secretion in the kidney has not been reported. C1QTNF3 encodes C1q/TNF-related Protein-3, which is an adipokine, and, thus, has been studied extensively in adipose tissue and cell lines [36]. However, it is rarely reported in the kidney. CGREF1, which is also known as CGR11, encodes hydrophobestin. This protein has been reported to be secreted in MtT/S cell culture [37]. SECTM1B encodes secreted and transmembrane protein 1. This protein shows a perinuclear Golgi-like pattern; its N-terminal domain was reported to be secreted in peripheral blood leukocytes and breast cancer cell lines [38]. However, there are no reports about this gene in the kidney.

As shown by semi-quantitative PCR, both LRG1 and SERPINA3N are highly expressed in both liver and adipose tissue (Fig 1). However, they are only reported to be secreted in liver. Leucine-Rich Alpha-2-Glycoprotein (LRG1) is a plasma glycoprotein primarily produced in the liver [39]. It is also synthesized and secreted by ovarian cancer cells [40]. SERPINA3N is one isoform of murine α-1 antichymotrypsin (ACT), which has also been reported to be highly expressed in the liver. ACT is primarily synthesized and secreted by hepatocytes and released in plasma. In addition, SERPINA3N is reported to be secreted in Sertoli cell cultures [41].

Among the remaining 6 genes, DHRS7C is located in the sarcoplasmic reticulum of the heart and skeletal muscle [42], although it is predicted to be secreted by WoLF PSORT. The other 5 genes—CTLA2A in the lung, MUP19 in the liver and WFDC15B, SERPINA1F and DEFB29 in the kidney have not been reported to be secreted in currently available publications.

### Identification of Alternative Splicing of CTLA2A and SERPINA1F

**Fig 2:**
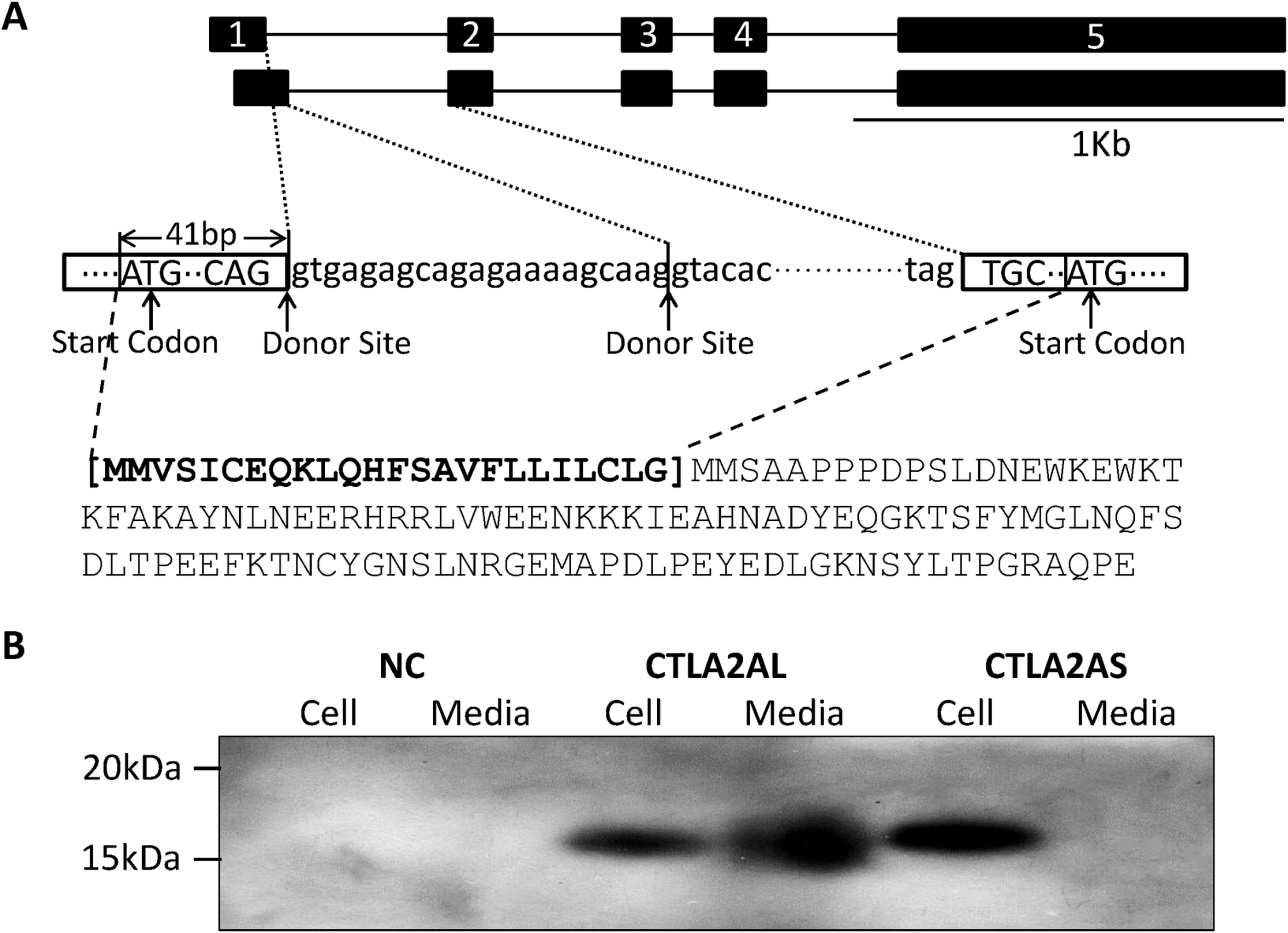
Different isoforms of CTLA2A and their expression in HEK293 cell culture. **A,** alternative splicing of CTLA2A that leads to change in protein sequence. **B,** cell lysates and conditioned medium from HEK293 cells transiently transfected with HA-tagged murine expression vector of long isoform (CTLA2AL) and short isoform (CTLA2AS) and empty pcDNA3.1 vector as negative control (NC) were detected using Western blotting.

**Fig 3:**
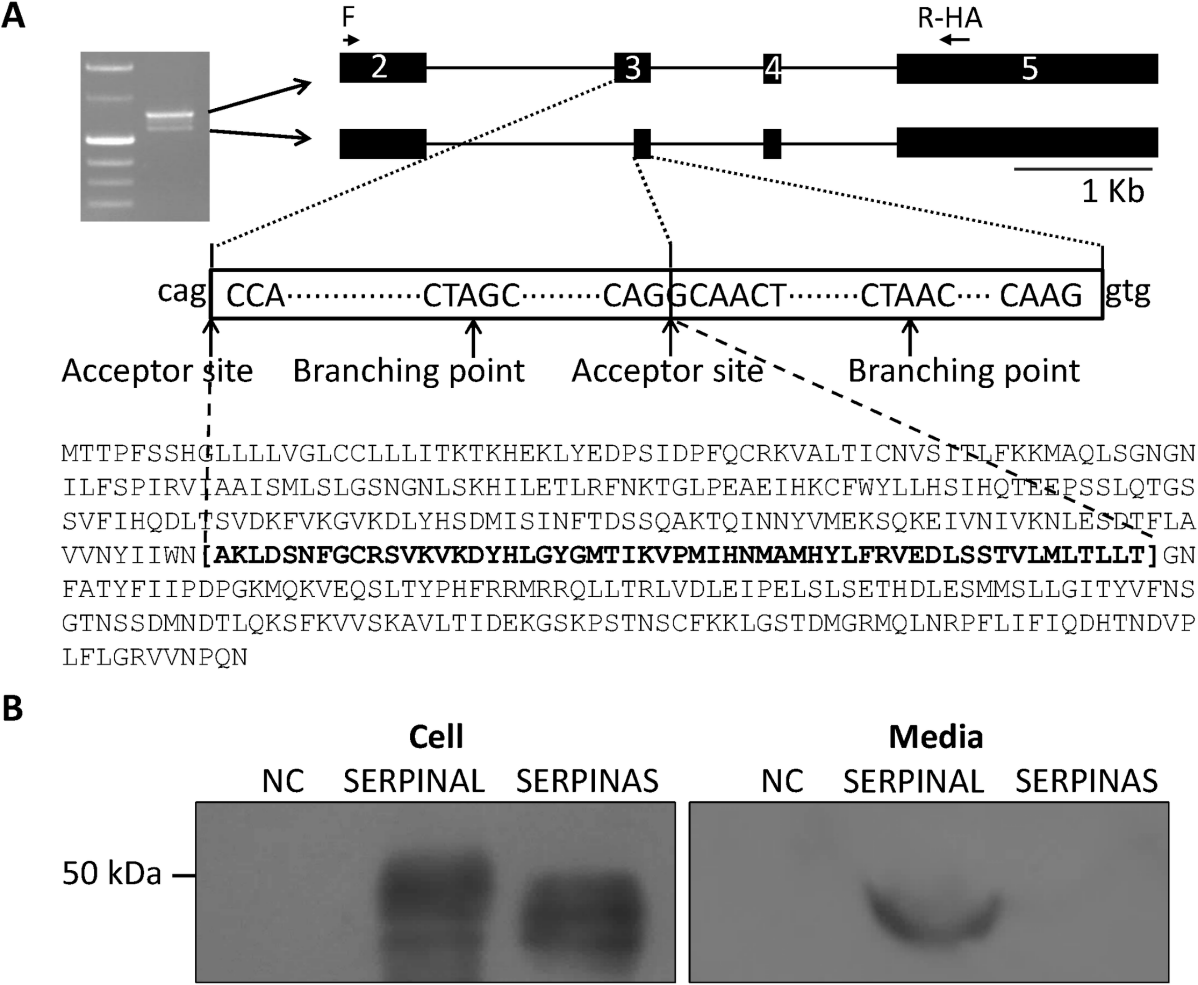
Different isoforms of SERPINA1F and their expression in HEK293 cell culture. **A,** alternative splicing of SERPINA1F that leads to double bands in gel electrophoresis of PCR product and change in protein sequence. **B,** cell lysates and conditioned medium from HEK293 cells transiently transfected with HA-tagged murine expression vector of long isoform (SERPINAL) and short isoform (SERPINAS) and empty pcDNA3.1 vector as negative control (NC) were detected using Western blotting.

To detect whether proteins translated by the 5 genes are secreted or not, the open reading frame (ORF) of each gene was amplified by a forward primer at the start codon and a reverse primer linked with HA-tag sequence. Alternative splicing of CTLA2A (NM_007796 and NM_001145799) and SERPINA1F (NM_026687 and NM_001164742) were identified in GenBank. As shown in Fig 2A, there are alternative start codons near the end of exon 1 and beginning of exon 2, whereas the alternative start codon in exon 2 only occurs together with the alternative donor site in intron 1, resulting in a loss of 24 N-terminal amino acids. However, the amount of this short isoform is too small to be amplified by PCR. Unlike CTLA2A, the alternative splicing of SERPINA1F occurs within an exon rather than an intron. The ORF of SERPINA1F starts from the second base pair in the second exon and ends at 186 bp in exon 5. As shown in Fig 3A, in-frame alternative splicing occurs in the region transcribed from exon 3, which contains an alternative acceptor site, resulting in deletion of 165 nt in mRNA and 55 amino acids in protein.

The alternative splicing in both genes fits the classical splicing model outlined by Keller and Noon [43] and Padgett et al. [44]. In the classical model, the consensus sequence at the donor site should usually be AG at the 3’ end of an exon followed by GT at the beginning of the adjacent intron. This feature is located not only at the 5’ end of intron 1, but also 21 bp from the 5’ end of intron 1 of CTLA2A, making alternative splicing feasible. On the other hand, the consensus sequence at the acceptor site is usually AG at the 3’ end of an intron followed by G at the beginning of adjacent exon. The sequence CTA/GAC/T at the branching point should be 20–50 base pairs from the 3’ splice site. These features are located not only in the 3’ end but also in the middle area of exon 3 in Serpina1f, which facilitates the alternative splicing.

### Detection of Novel Secretory Genes in HEK293 Cell Culture

**Fig 4:**
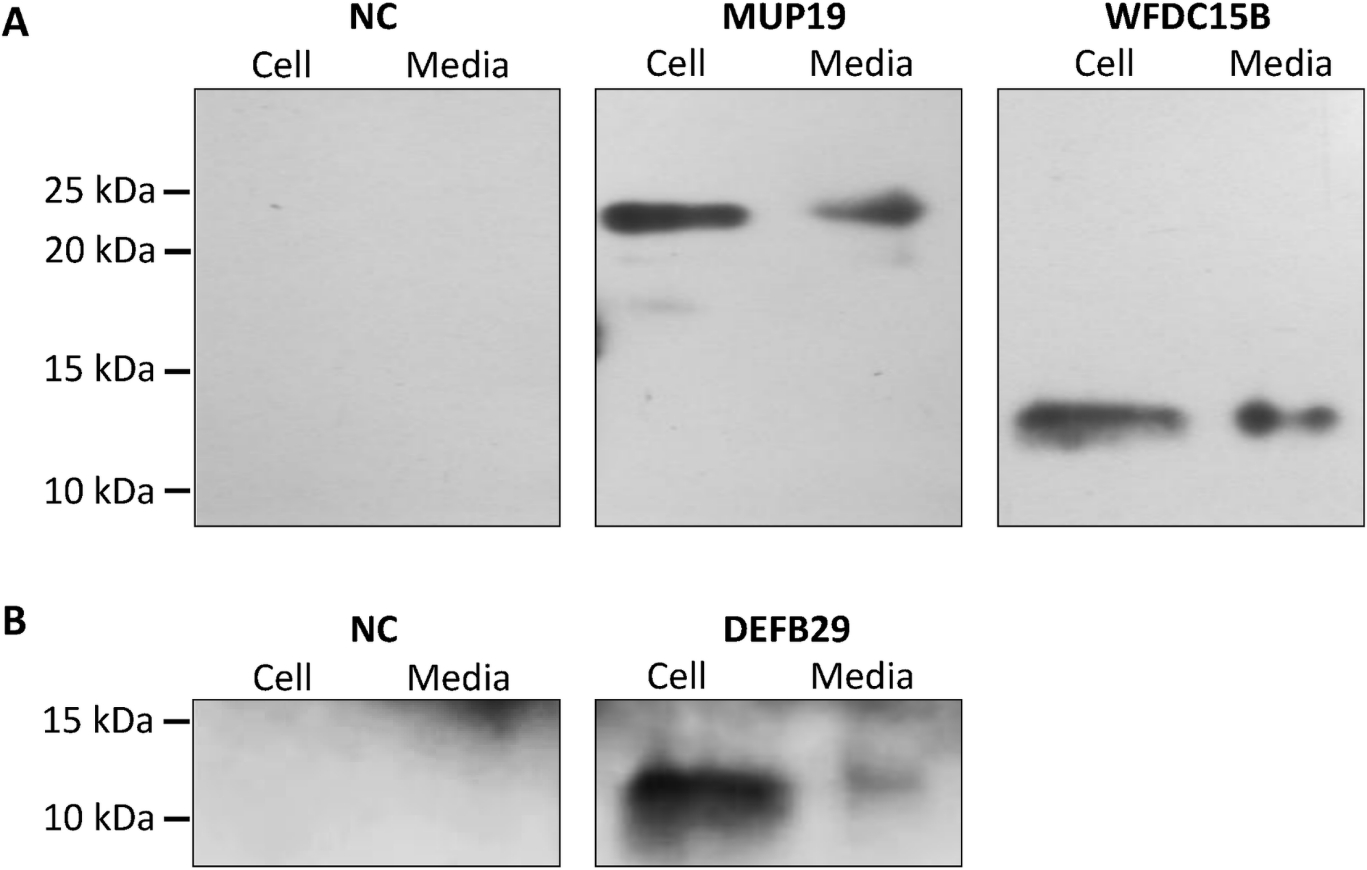
Detection of protein expression of MUP19, WFDC15B and DEFB29 in HEK293 cell culture. **A,** Cell lysates and conditioned medium from HEK293 cells transiently transfected with HA-tagged murine expression vector of MUP19 and WFDC15B and empty pcDNA3.1 vector as negative control (NC) were detected using Western blotting. **B,** Cell lysates and conditioned medium from HEK293 cells transiently transfected with HA-tagged murine expression vector of DEFB29 and empty pcDNA3.1 vector as negative control (NC) were detected using Western blotting.

Western blotting demonstrates that CTLA2A with full-length amino acids is secreted as it was detected in both cell lysate and conditioned media. However, the short isoform with deletion of N-terminal amino acids is not secreted as predicted by WoLF PSORT (Fig 2B), because it lacks signal peptide. For SERPINA1F, the long isoform is detected in both cell lysate and conditioned media, indicating that it is secreted outside the cells. However, the short isoform, which lacks 55 amino acids in the middle region of the protein, is not secreted, because it was only detected in cell lysate (Fig 3B). WFDC15B, DEFB29 and MUP19, which do not have alternative splicing, are also detected in both cell media and lysate (Fig 4), indicating their secretion out of cells. Finally, for the cells transfected with empty vectors, no protein was detected. This validates the successful insertion of target genes into the vector and expression of target genes in HEK293 cells.

### GEO Data Analysis of Novel Secretory Genes

For the 5 novel secretory genes, there are few reports about their temporal and spatial expression, as well as function, in the tissues where they are highly expressed. In order to understand potential functions of these genes, gene expressions were compared between different age groups, different tissue sections and different treatment groups or genetic lines by analyzing GEO profiles.

**Fig 5:**
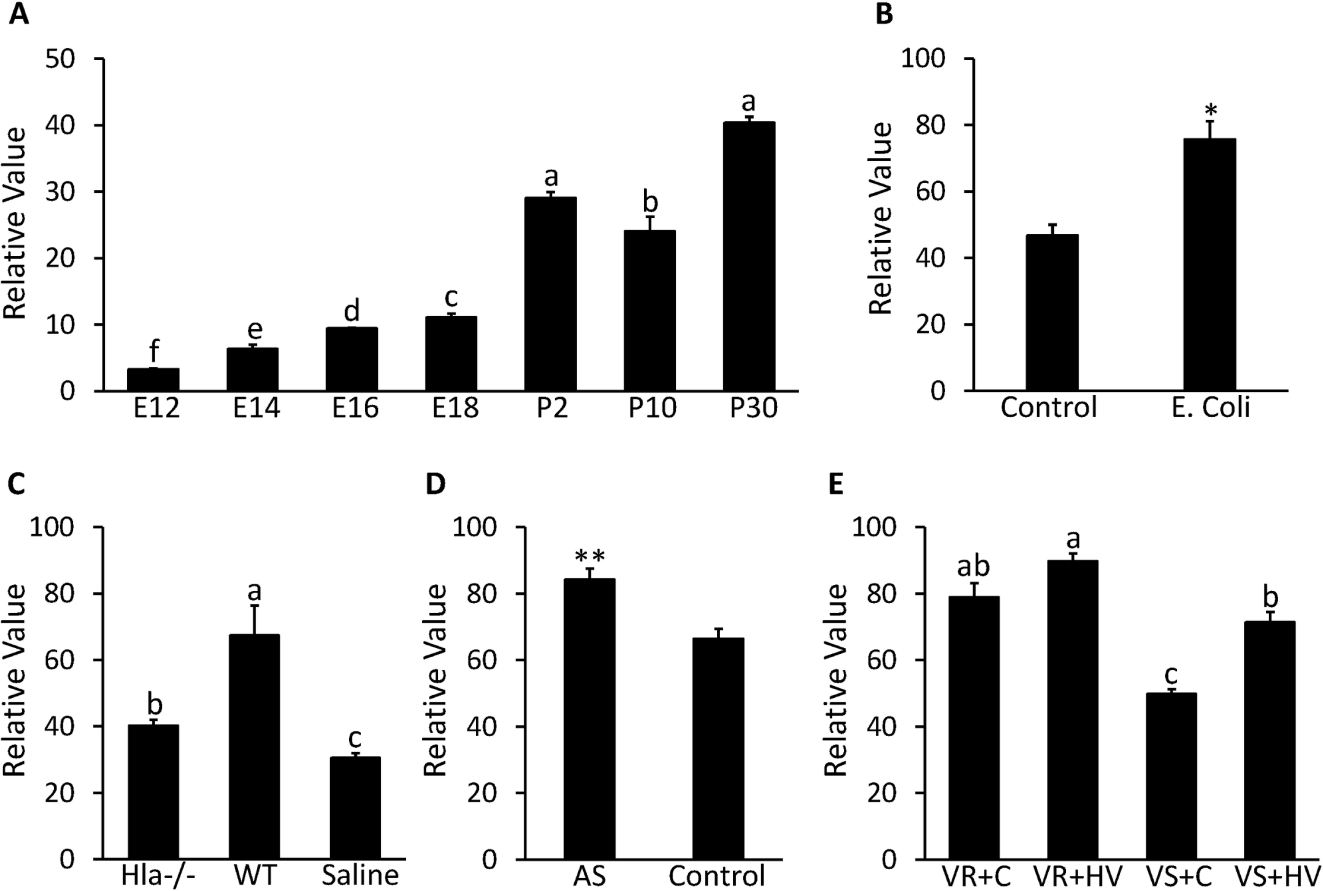
Expression profile in lung for CTLA2A in microarray DataSets obtained from NCBI website. **A,** Expression in mice on embryonic day 12 (E12), 14 (E14), 16 (E16) and 18 (E18) and postnatal d 2 (P2), 10 (P10) and 30 (P30) in GDS3950 (n = 2 per time point). **B,** Expression in mice infected by *E*.*coli* and control group in GDS4583 (n = 3 per group). **C,** Expression in mice after 24-h infection by *S*.*aureus* deficient in alpha-hemolysin (Hla-/-) and wild type S.aureus (WT) with saline treated mice as control group (n = 3 per group). **D,** Expression in mice after 90-d exposure to 50 ppm arsenate (AS) with untreated mice as control in GDS4914 (n = 5 per group). **E,** Expression in resistant rat strain (VR) to ventilator-associated lung injury (VALI) and sensitive rat strain (VS) to VALI under treatment of high tidal volume ventilation (HV) with untreated mice as control (C) group in GDS2709 (n = 3 per group). Each bar represents mean±SEM. Statistical difference is indicated by different letters (P<0.05), * (P<0.05) and ** (P<0.01) above the bars.

Expression of CTLA2A in the lung seems to be affected by bacterial infection and other injuries. As can be seen from Fig 5A, GDS3950 shows that CTLA2A expression exhibits an increasing trend during development of the mouse lung except for a slight decrease on postnatal d 10. In particular, there is a dramatic increase in expression after the mouse is born (P<0.0001). GDS4583 shows that expression of CTLA2A in the lungs of C57BL/6 mice increased 1.6 times (P<0.05) after intratracheal instillation of *Escherichia coli* (*E*. *coli*) (Fig 5B). In another case, GDS4582 suggests that CTLA2A expression is promoted more than 2 times (P<0.05) 24 h after infection by wildtype *Staphylococcus aureus (S*. *aureus)*. On the other hand, deletion of Alpha-hemolysin (Hla)—an essential lethal factor secreted by *S*. *aureus-* reduced expression of CTLA2A to a medium level (Fig 5C). In GDS4914, expression of CTLA2A in the lungs of C57BL/6 mice increased 1.3 times (P<0.01) after exposure to 50 ppm of arsenate for 90 d compared to the control group (Fig 5D). A similar change occurred in GDS2709 as well (Fig 5E). In a rat strain resistant to ventilator-associated lung injury (VALI), expression of CTLA2A is significantly higher (P<0.05) than that in a strain sensitive to VALI under the same treatment. In the VALI sensitive strains, high tidal volume ventilation induced expression of CTLA2A 1.4 times (P<0.001) compared to the control group.

**Fig 6:**
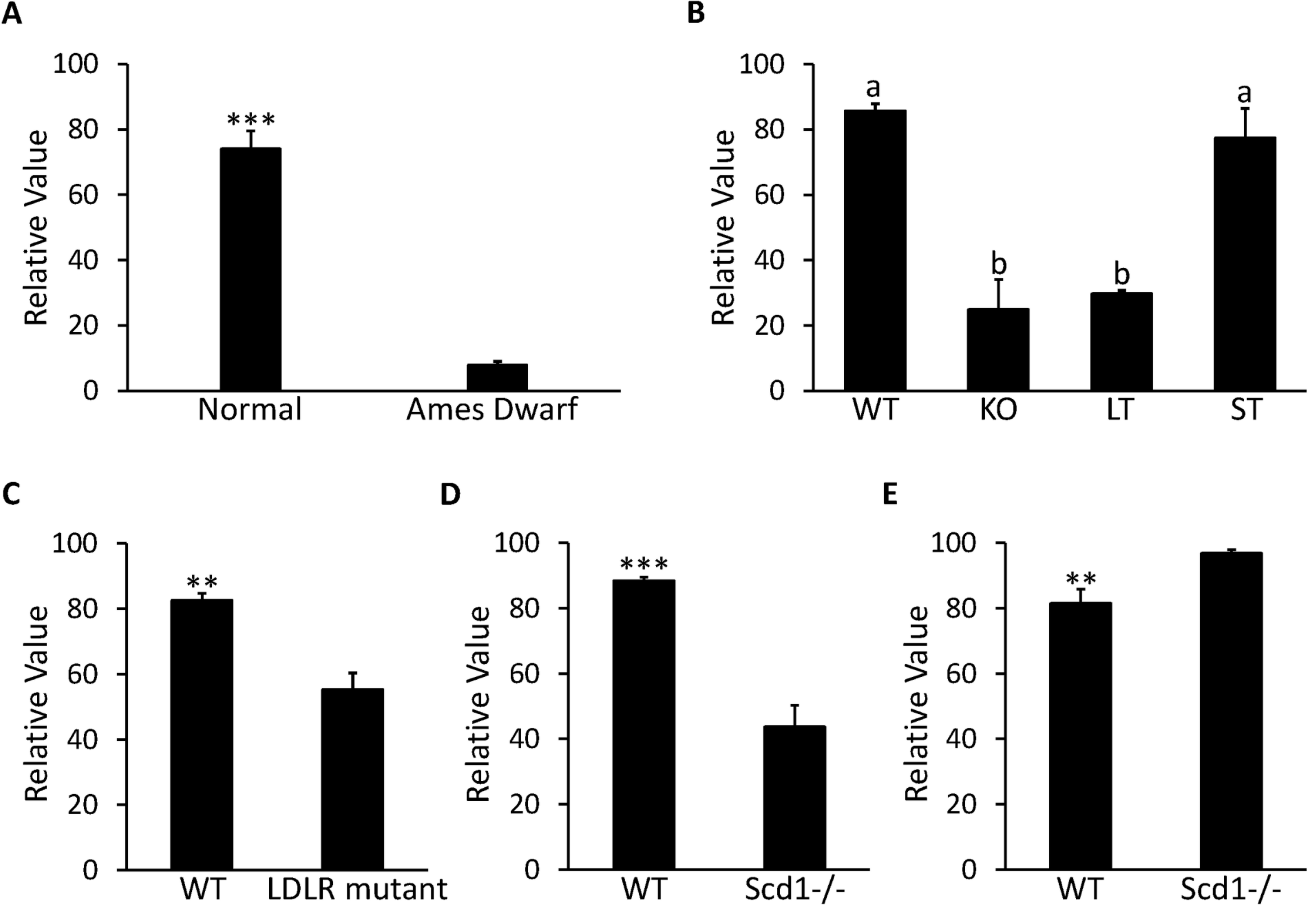
Expression profile in liver for MUP19 in microarray DataSets obtained from NCBI website. **A,** Expression in Ames Dwarf mice (n = 8) and normal mice (n = 7) with free access to standard food in GDS1261. **B,** Expression in 42-d old mice with growth hormone receptor knocked out (KO), truncated at amino acid residue 569 (ST) and 391 (LT) with wild type (WT) mice as control group in GDS1053 (n = 3 per group). **C,** Expression in C57BL/6 mouse with null mutant low-density lipoprotein receptor (LDLR) under the treatment of high fat diet for 12 wk along with WT mice as control group in GDS279 (n = 3 per group). **D,** Expression in mice with stearoyl-CoA desaturase 1 deficient mutants (Scd1-/-) under treatment of low-fat, high-carbohydrate diet along with WT mice as control group in GDS1517 (n = 5 per group). **E,** Expression in 6-wk Scd1-/- mice fed with chow diet along with WT mice as control group in GDS1374 (n = 5 per group). Each bar represents mean±SEM. Statistical difference is indicated by different letters (P<0.05), ** (P<0.01) and *** (P<0.001) above the bars.

The correlation between MUP19 and the GH/IGF-1 signaling pathway is demonstrated by GDS1261 and GDS1053. According to GDS1261, expression of MUP19 was reduced more than 90% in Ames dwarf mice with mutant pituitary specific transcription factor 1 (PIT-1)—one key regulator in the GH/IGF-1 signaling pathway (Fig 6A). In GDS1053, knockout of the growth hormone receptor (GHR) led to a 60% decrease in MUP19 expression (P<0.001) in the liver of 42-d old mice. In addition, when the GHR was truncated at residue 596, there was not a significant decrease in MUP19; when GHR was truncated at residue 391, the decrease in MUP19 became 65% (P<0.0001) compared to the wild type mice (Fig 6B). Expression of MUP19 is also affected by deficiency of low density lipoprotein (LDL) receptors. In GDS279, expression of MUP19 in the liver was repressed by 33% in C57BL/6 mice lacking LDL receptors compared to the normal mice after high fat diet treatment for 12 wk (Fig 6C). The correlation between MUP19 and Stearoyl-CoA desaturase 1 (SCD1) seems to be affected by food intake. In GDS1517, expression of MUP19 decreased 50% in livers of mice with SCD1 deficiency compared to wild type mice under treatment of a low fat, high carbohydrate diet (P<0.001, Fig 6D). However, in GDS1374, where the SCD1-/- mice were fed a chow diet, the expression of MUP19 was increased 1.2 times compared to the wild type mice (P<0.01, Fig 6E).

**Fig 7:**
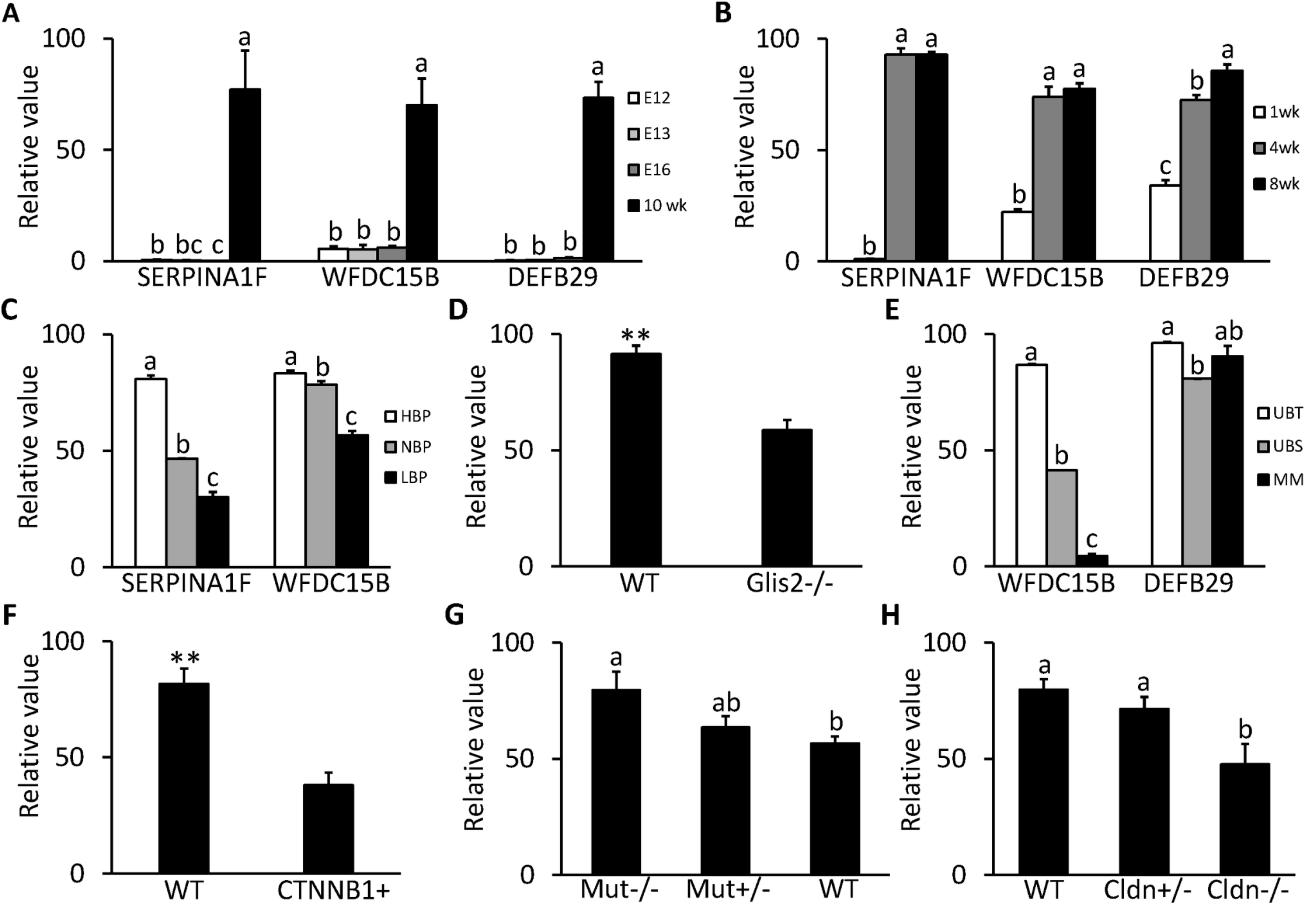
Expression profile in kidney for kidney specific genes in microarray DataSets obtained from NCBI website. **A**, Expression of SERPINA1F, WFDC15B and DEFB29 in mice on embryonic d 12 (E12), 13 (E13) and 16 (E16) and postnatal 10 wk in GDS2030/2031 (n = 2 per time point). **B**, Expression of SERPINA1F, WFDC15B and DEFB29 in postnatal C57BL/6 mice at 1, 4 and 8 wk of age in GDS4316 (n = 5 per time point). **C**, Expression of SERPINA1F and WFDC15B in hypertensive (HBP, n = 5), normotensive (NBP, n = 5) and hypotensive (LBP, n = 4) mice strain in GDS3675. **D**, Expression of WFDC15B in mice with null mutant GLIS family zinc finger 2 (GLIS2-/-) and wild type (WT) mice in GDS2817 (n = 3 per group). **E**, Expression of WFDC15B and DEFB29 in ureteric bud tips (UBT) and stalks (UBS) and metanephric mesenchyme (MM) from embryonic d 12 mice in GDS1583 (n = 2 per section). **F**, Expression of WFDC15B in mice with b-catenin overexpression (CTNNB1+) and WT mice on embryonic d 12 in GDS4449 (n = 3 per group). **G**, Expression of DEFB29 in homozygous mice for mutant methylmalonyl-CoA mutase (Mut-/-), heterozygous mice (Mut+/-) and WT mice in GDS4839 (n = 4 per group). **H**, Expression of DEFB29 in WT mice (n = 4), heterozygous mice (Cldn+/-, n = 3) and homozygous mice for mutant claudin 16 (Cldn-/-, n = 4) in GDS 3612. Each bar represents mean±SEM. Statistical difference is indicated by different letters (P<0.05) and ** (P<0.01) above the bars.

SERPINA1F, WFDC15B and DEFB29 are all highly expressed in the kidney. From GDS2031/2030 (Fig 7A) and GDS4316 (Fig 7B), it can be seen that the expressions of all 3 genes were at a very low level during the embryonic and neonatal stages and only increased after the mouse was born and stayed at a high level or kept increasing after postnatal 4 wk, indicating that these 3 genes are important for normal function of the developed kidney.

SERPINA1F encodes one isoform of serine peptidase inhibitor, clade A, member 1 (SERPINA1). In GDS3675, it seems that expression of SERPINA1F is positively related to blood pressure in the kidney (Fig 7C). Expression in the hypertension mouse strain is about 2 times that of normotension mice (P<0.0001), while the expression in the hypotension mouse strain dropped 30% compared to that of normotension mice (P<0.0001).

As was the case for SERPINA1F, expression of WFDC15B in the kidney is also positively correlated with blood pressure as shown in GDS3675 (Fig 7C). Expression in the hypertension mouse strain is about 1.1 times that of normotension mice (P<0.05), while expression in the hypotension mouse strain also dropped 30% compared to normotension mice (P<0.0001). In GDS2817, expression of WFDC15B is downregulated by 36% (P<0.01) in the kidney of mice with severe kidney atrophy and fibrosis caused by loss of the GLIS2 gene compared with normal mice (Fig 7D). In GDS1583, expression of WFDC15B at the ureteric bud tip is 2 times and 10 times higher respectively than in the ureteric bud stalk and the metanephric mesenchyme in the embryonic kidney (Fig 7E). In GDS4449, in kidneys of 12 d mouse embryos with defective ureteric branching morphogenesis and nephrogenesis due to overexpression of β-catenin, WFDC15b was downregulated by 54% (P<0.01) compared to the wild type group (Fig 7F).

As was the case for WFDC15B, DEFB29 shows higher expression in the ureteric bud tip than in the ureteric bud stalk (P<0.0001) in the mouse embryonic kidney in GDS1583, but its expression in the metanephric mesenchyme is not significantly lower than in the ureteric bud tip (Fig 7E). In GDS4839, expression of DEFB29 is prompted by mutation of methylmalonyl-CoA mutase (MUT). In the homozygous mutant mice, expression of DEFB29 increased 1.4 times compared to that in wild type mice (P<0.05). In the heterozygotes, expression of DEFB29 also showed an increasing tendency (Fig 7G). In GDS3612, another interesting pattern occurs in expression of DEFB29, as it seems positively correlated with expression of functional Claudin 16 (CLDN16). In mice with the homozygous mutant CLDN16, the expression of DEFB29 is 40% less than in wild type mice (P<0.05); in the heterozygous mice, expression of DEFB29 also shows a decreasing trend (Fig 7H).

## Discussion

In this study, we successfully applied the powerful approach established by Song et al. [10] to select novel tissue-specific genes in the mouse. Among the 31 tissue-specific genes selected by microarray data analysis and semi-quantitative PCR analysis, there are 3 main categories: i) genes that were reported by previous publications to be secretory genes in the tissues in which we detected high expression; ii) genes that were reported by previous publications to be secretory genes, but secretion had not been reported in the tissues in which we detected high expression; and iii) genes that were not previously reported to be secretory genes.

### Lung-Specific Genes

Among the 11 lung specific genes, CHIL4 (Chitinase-like protein 4), LYZ1 (lysozyme) and SEMA3G (semaphorin 3G) belong to category i. CHIL4 is a member of the Chitinase-like proteins, which are indicators of inflammation and cancer in humans [45]. CHIL4 itself is also suggested to play an important role in airway inflammation [46]. LYZ1 is the primary antibacterial component secreted by submucosal glands in tracheobronchial airways in lung [21]. SEMA3G is a member of Class 3 semaphorins, which are chemorepellants for growing neurons and axons [47]. However, there is also accumulating evidence for involvement of some semaphorins in the immune system [48]. Therefore, SEMA3G may also be involved in both the nervous and immune systems in the lung.

NDNF (neuron-derived neurotrophic factor), NPNT (nephronectin), PCOLCE2 (procollagen C-endopeptidase enhancer 2), LGI3 (leucine-rich glioma inactivated 3), EGFL6 (epidermal growth factor-like protein 6), HILPDA (hypoxia inducible lipid droplet-associated protein), and SPON1 (F-spondin) belong to category ii. They are reported to be secreted in some cell cultures or tissues, but not to be secreted or specific to the tissues in which we detected high expression. NDNF was recently reported to be a regulator that promotes endothelial cell function and its secretion is reported to be stimulated by hypoxia in the vascular system [49]. So, it may play a similar role in the lung. NPNT is a ligand of integrin subunit α8β1, which is an important regulator that promotes branching morphogenesis in the lung [50] and kidney [51]. The interaction between NPNT and integrin α8 has proved to be important for kidney development [51]. Therefore, NPNT may play an important role in lung morphologenesis. PCOLCE2 was reported to be highly expressed in the adult human heart rather than in the lung. It is an enhancer for sufficient procollagen processing that is required for myocardial collagen deposition in the adult human heart [52]. Thus, it may also play the same role in the extracellular matrix of lung. LGI3 is a member of the secreted leucine-rich repeat LGI family. This protein family was reported to be crucial for development and function of the nervous system [53]. LGI3 has been proven to induce neurite outgrowth in the brain [19] and promote metabolic inflammation in adipose tissue [54]. Therefore, it may also be involved in the nervous and immune systems in the lung. EGFL6 was reported to promote endothelial cell migration and angiogenesis [20]. Therefore, it may be important for the endothelium of vasculature, including that in pulmonary circulation. HILPDA was reported to be a target protein of hypoxia-inducible factor 1 (HIF-1) and upregulated by hypoxia [55], indicating that it is involved in metabolism regulation and oxygen delivery in the lung. SPON1 was reported to inhibit human umbilical vein endothelial cell migration and angiogenesis [24]. Therefore, SPON1 may be important in the stabilization of endothelial cells in the mature lung as well.

CTLA2A (cytotoxic T lymphocyte-associated protein 2 alpha) belongs to category iii. It is mostly expressed in cytotoxic T lymphocytes, and its expression is induced upon lymphocyte activation [56]. Therefore, it is tightly correlated with immunological response. Because of the massive surface of lung epithelium and continuous exposure to external environment, the lung has many T lymphocytes to regulate host defenses against viruses, fungal pathogens and bacteria [57]. Therefore, high expression of CTLA2A in the lung may be essential for normal T cell response and pulmonary host defense. As indicated by the cell culture and Western blot analysis, alternative splicing of CTLA2A, which can barely be detected by PCR, leads to a non-secretory protein without signal peptide. Therefore, the secretion of this protein is predominant with only a tiny amount maintained inside the cell. In GEO data analysis, expression of CTLA2A increased dramatically after birth due to exposure to the external environment, after infection with *E*.*coli* and wild type *S*. *aureus*, and after long-term exposure to 50 ppm of arsenate and high tidal volume ventilation. On the other hand, its expression is also moderately elevated in a rat strain that is resistant to VALI and in mice infected by *S*. *aureus* lacking lethal factor. All of these data suggest that CTLA2A plays an important role in protecting the lung from harmful invasion.

### Liver-Specific Genes

All of the liver-specific genes except MUP19 belong to category i, since the secretion of their products has been reported in the liver. SERPINA1C (Serpin Peptidase Inhibitor, Clade A, member 1C) is one member of SERPINA1. Liver injury triggered by accumulation of mutant SERPINA1 in ER of hepatocytes is the most common genetic cause of hepatic disease in children [58]. CPB2 (Carboxypeptidase B2) is secreted to plasma from the liver and is upregulated during the inflammatory response and inhibits fibrinolysis and inactive inflammatory peptides after activation by the thrombin-thrombomodulin complex on the vascular endothelial surface [59]. ITIH1 and ITIH3 are 2 heavy chains of ITI. ITI is also a plasma protein secreted by the liver and is well-known for its anti-inflammatory function and stimulatory function in endothelial cells [60]. C8B is a subunit of complement component 8 (C8) protein. C8 is a component of the membrane attack complex (MAC), which plays a key role in the innate and adaptive immune system by mediating cell lysis [61]. KLKB1 (kallikrein B) is also a plasma protein secreted by the liver. It can stimulate release of neutrophil elastase release during blood coagulation, and also participates in production of plasmin from plasminogen, the kallikrein-kinin system, the renin-angiotensin system and the alternative complement pathway [62].

MUP19 (major urinary protein 19) belongs to category iii, although according to its name, it belongs to the MUP family, which is a group of lipocalins secreted from the liver and excreted to urine after circulation [63]. Its secretion and function have not been reported. Therefore, we can only speculate as to its function based on GEO data analysis and the function of other members of the family. From GEO data analysis, we predict that MUP19 is positively regulated by growth hormone (GH), since mutation in the PIT-1 gene causing reduced GH production and deletion of GHR, both dramatically reduced expression of hepatic MUP19. In addition, the intensified decrease in expression of MUP19 is also concordant with the exaggerated truncation of GHR [64]. In addition, decrease of MUP19 in mice with LDL receptor deficiency indicates expression of MUP19 is regulated by lipid homeostasis in the liver, because mice with LDL receptor deficiency cannot intake lipids to the liver from blood circulation [65]. Finally, we predict that MUP19 is a protein regulated by lipid metabolism in liver due to modulation of its expression by dietary treatment in mice lacking SCD1. Because SCD1 is a key enzyme in lipid metabolism that catalyzes the de novo synthesis of monounsaturated fatty acids (MUPA), which are components of triglycerides, its ablation impairs not only MUFA synthesis but also lipid metabolism in the liver induced by low-fat, high-carbohydrate diets [66]. Therefore, the expression of MUP19 is reduced. However, when enough dietary MUPA can be obtained from food, there is no compensatory increase in fatty acid synthesis. Instead, the activation of lipid oxidation and reduction in triglyceride synthesis produced an anti-obesity effect [67]. Therefore, the expression of MUP19 increased. The reduction of MUP19 expression by low-fat, high-carbohydrate diets and induction of its expression by a chow diet indicates that MUP19 may play a positive role in lipid metabolism promotion and may be related to lipid deposition in the liver. This role is similar to that of MUP1, which has been extensively studied. Liver-specific overexpression of MUP1 significantly reduced triglyceride levels in livers, and chronic treatment with MUP1 also reduced plasma lipids in db/db mice [68].

### Heart/Muscle-Specific Genes

The 2 heart-specific genes, FNDC5 (fibronectin type III domain-containing protein 5) and IL15 (interleukin 15) are in category i. The secreted protein of FNDC5—irisin is induced after physical exercise, and increases caloric expenditure by converting white adipose tissue to brown adipose tissue, and, thus, opposes the formation of atheromata [69]. IL15 is a widespread cytokine regulating immune response. It can stimulate the proliferation of T cells, and promote development and function of natural killer cells. However, according to the GEO data analysis and semi-quantitative PCR analysis, IL15 is expressed more in the heart than in the other 5 tissues, indicating that it may have a special function in the heart. It has been proven that elevated circulating levels of IL15 can reduce body fat significantly and inhibit obesity [70]. Therefore, high expression of IL15 may be helpful in preventing excessive lipids in the cardiovascular system. GPC1 (glypican-1) is an important protein in both skeletal and cardiac muscle, since it is specifically expressed in both the muscle and heart. As suggested by Velleman et al. [71], GPC1 promotes myogenic satellite cell proliferation and responsiveness of fibroblast growth factor 2 (FGF2), which is a stimulator of muscle cell proliferation and an inhibitor of differentiation.

### Kidney-Specific Genes

Except for SERPINA1F, WFDC15B and DEFB29, the other 4 kidney-specific genes belong to category ii because there are no publications about their secretion in kidney. However, the high expressions we detected in mouse kidney suggest their potential functions in the kidney. SPINK3 (serine peptidase inhibitor, kazal type 1) is highly expressed in the pancreas and has mainly been studied in the pancreas and sex glands. It is known as a protease inhibitor, a growth factor for acinar cells in the pancreas, and an inhibitor of calcium uptake on the sperm head [72]. Therefore, SPINK3 may also function as a regulator of calcium transport or as a growth factor in the kidney. C1QTNF3 (c1q and tumor necrosis factor related protein 3) is specifically expressed in cartilage and kidney in adult mice [73]. It is known as an adipokine opposing leptin [36] and an enhancer for skeletal development [73]. However, its function in the kidney is still unclear. CGREF1 (cell growth regulator with EF-hand domain 1) is a protein that mediates cell adhesion [37] and is an inhibitor of cell growth [74]. SECTM1B (secreted and transmembrane 1b) is one isoform of SECTM1. It is a chemoattractant for monocytes [75] and a stimulator for T cell activation [76], and, thus, may play an important role in the kidney immune system.

SERPINA1F, WFDC15B and DEFB29 belong to category iii. SERPINA1F was reported to be predominantly expressed in the epididymis of male mice and is reduced after castration and recovered by administration of testosterone [77]. However, our study indicates that SERPINA1F is also highly expressed in the kidney, although there are no reports concerning the expression and function of this gene in the kidney. SERPINA1F encodes one isoform of serine peptidase inhibitor clade A member 1 (SERPINA1). Expression of SERPINA1 is up-regulated by acute kidney injury and can protect injured proximal tubule cells by reducing activity of neutrophil elastase [78]. Therefore, SERPINA1F may also play a role in protection of the kidney. The increase of SERPINA1F expression in a hypertension mouse strain and decrease in a hypotension mouse strain indicates that it may be involved in regulation of response to renovascular blood pressure. The increase in SERPINA1 excretion has been reported in patients with arterial hypertension [79]. One patient with SERPINA1 deficiency also developed uncontrolled hypertension [80]. Therefore, SERPINA1F may play a critical anti-hypertension role in mice as does AAT in humans. This may also be attributed to its role in kidney protection since renal hypertension usually causes vascular injury and inflammatory response in the kidney. As shown in cell culture and Western blotting, the peptide translated by alternative splicing lacking 55 residues seems to be retained inside the HEK293 cells rather than secreted. This situation is similar to that of soluble secreted endopeptidase (SEP) and cholesteryl ester transfer protein (CETP). In SEP, deletion of 23 residues following the transmembrane domain at the beginning of the luminal domain resulted in retention of the protein in ER [81]. In CETP, deletion of 60 amino acids in the central region also resulted in its retention within ER [82]. According to prediction of the transmembrane protein with the TMHMM server (http://www.cbs.dtu.dk/services/TMHMM/), the deleted region in SERPINA1F also closely flanks one transmembrane domain. Therefore, the short peptide translated by alternative splicing may also stay in the ER and the retention may be also mediated by the luminal domain as SEP. In addition, inhibition of secretion of full-length protein by the short protein for CETP through complex association also provides a clue for future study of the association between the 2 isoforms of SERPINA1F.

WFDC15B encodes single whey acidic protein (WAP) motif protein 1 (SWAM1) in the mouse, which is an antibacterial protein. Its expression was detected in the kidney and epididymis and its antibacterial property has also been observed for *E*. *coli* and *S*. *aureus* [83]. Therefore, it may participate in the immune system. GEO profiles indicate that WFDC15B is important for both the health of the mature kidney and development of the embryonic kidney, because its expression was reduced significantly after the occurrence of severe kidney atrophy and defective ureteric branching morphogenesis. In addition, high expression of WFDC15B in ureteric bud tip cells may indicate its contribution to development of nephrons as a secretory factor from the ureteric bud tip, because the ureteric bud tip cells play an important role in inducing conversion of metanephric mesenchyme into nephrons by secreted factors [84]. In addition, WFDC15B may be also associated with blood pressure as it shows the same expression pattern as SERPINA1F in hypertension and hypotension mouse strains compared with the normotension strain.

DEFB29 (defensin, beta 29) is one member of the β-defensin family, which is a group of small antibiotic proteins involved in host defense by disrupting the cytoplasmic membrane of microorganisms [85]. Therefore, DEFB29 may contribute to the immune response of infections and inflammatory disease. GEO data analysis indicates that expression of DEFB29 is increased concordantly with the loss of methylmalonyl-CoA mutase (MUT). Because MUT is an enzyme that mediates metabolism of carbon skeletons through the Krebs cycle, deficiency of MUT usually causes increased oxidative stress in the renal tubule, which leads to chronic tubulointerstitial nephritis [86]. Therefore, the increase in DEFB29 may reflect the immune response caused by renal tubular dysfunction. In addition, DEFB29 may also be related to renal Ca^2+^ and Mg^2+^ homeostasis, because its expression decreases concordantly with the loss of Claudin 16 (CLDN16). As reported by Will et al. [87], CLDN16 is a critical regulator of Ca^2+^ and Mg^2+^ transport in the thick ascending loop of Henle. Finally, DEFB29 may play an important role in development of the ureteric bud and metanephric mesenchyme since it is highly expressed in both structures in embryonic kidneys.

### Adipose and Liver-Specific Genes

The 2 adipose and liver-specific genes, LRG1 (leucine-rich alpha-2-glycoprotein) and SERPINA3N (serpin peptidase inhibitor, clade A, member 3N) belong to category i, but they are only reported to be secreted from liver to plasma. LRG1 in plasma can sequester cytochrome C (Cyt C) released by apoptotic cells and protect lymphocytes from the toxic effects of Cyt C [88]. In addition, LRG1 can promote angiogenesis by regulating transforming growth factor β (TGF-β) signaling [89]. Adipose stromal cells have been reported to alleviate tissue damage and their secreted media has also been proven to protect neuronal cells from apoptosis [90]. In addition, preadipocytes also become relatively resistant to apoptosis during differentiation [91]. Therefore, LRG1 may be secreted in adipose tissue as a survival factor. In addition, angiogenesis is very important during adipogenesis, providing new vasculature to supply nutrients to and remove metabolic waste from growing and proliferating adipocytes [92]. Therefore, this evidence provides support for prospective studies on the role of LRG1 in adipose tissue.

SERPINA3N is one isoform of Serpin Peptidase Inhibitor, Clade A, member 3 (SERPINA3). This protein is a typical acute phase protein that is upregulated dramatically in response to inflammation. As an important inhibitor of leukocyte cathepsin G, SERPINA3 can prevent excessive or prolonged activity of cathepsin G that may lead to tissue damage at an inflammation site [93]. Therefore, secretion of SERPINA3 may also reflect function of adipose tissue related to regulation of metabolic inflammation and immune response.

From the above review of the selected genes, it can be noticed that functions of most genes in category ii are rarely studied in the lung and kidney; so, we can only speculate as to their possible functions in these tissues based on studies of their functions in other tissues. However, the possibility of special functions cannot be ruled out for these genes in the lung and kidney. Therefore, further research in these tissues is important for a thorough understanding of the functions of these genes. In general, our study developed a powerful approach to identify novel tissue-specific secretory genes by GEO data analysis, and to confirm it at the mRNA and protein levels by showing predominant expression of genes in specific tissues and secretion of these proteins. With the same strategy, more novel genes sharing common cellular locations, common physiological functions, or common pathological mechanisms can be explored in other animals, tissues and protein groups. In addition, the prediction of gene function by integrating valuable microarray data and the literature is also an efficient way to identify novel secretory genes related to different systems such as immune response, developmental regulation and homeostasis regulation.

## Supporting Information

S1 Table Primer sequences for semi-quantitative PCR. (DOCX)

S2 Table Primer sequences for amplification of insertions into expression vectors. (DOCX)
