# Supplementary material for: Identification of CTLA2A, DEFB29, WFDC15B, SERPINA1F and MUP19 as Novel Tissue-Specific Secretory Factors in Mouse
Source: PLoS One. 2015 May 6;10(5):e0124962. doi: 10.1371/journal.pone.0124962 (PMC4422522; doi:10.1371/journal.pone.0124962)
Supplement: S1 Table — (DOCX) [file pone.0124962.s001.docx]

**Table S1.** Primer sequences for PCR amplification

| Gene name | GenBank No. | Forward Primer Sequence (5’-3’) | Reverse Primer Sequence (5’-3’) |
| --- | --- | --- | --- |
| CTLA2A | NM_007796 | TCTTCAGAGACCGTGGACAACA | AATCAGGAGCCATTTCTCCTCTATT |
| LYZ1 | NM_013590 | CTCTGGGACTCCTCCTGCTTT | TGCTGACTGACAAGGGAGACTTT |
| SPON1 | NM_145584 | TGCTCCTCCCTCCTACTTCAGA | ACCAGTTGGAGTAGATGCAGGTTT |
| HILPDA | NM_023516 | TGTGAGCAGCCGTCTCTCA | ATGCTGCCTCCCGAGAAA |
| NDNF | NM_172399 | ATGAGGAACTTTTTCAGATGCAG | TGGTGGCATATACTTTGAAATGTG |
| LGI3 | NM_145219 | AGGACTGTCGCACCTGCAGTA | CTGGTAGATCTGGGTGAAGGAGAA |
| EGFL6 | NM_019397 | TGGAAGCTACTACTGCAAATGTCA | CAAAGAGCAAACAGAAGTTGCTTT |
| NPNT | NM_001287101 | CAAGGCAGTGAACAGCCTCTTTT | CACTTGCAGATGTAGCTCCCAAA |
| CHIL4 | NM_145126 | CTCCTGGCCATTGGAGGAT | CTGGTGGTCCAGCACTAACAGTA |
| SEMA3G | NM_001025379 | AACAAATGGAGCACTTTCCTCAA | AGCCCTGCCCAGCTCTT |
| PCOLCE2 | NM_029620 | AGTCTGGATTTATTGGCAGTGAA | CGAGACTGTGGCGTGCAA |
| CPB2 | NM_019775 | GAAACCTACTGTGGACTTTATCCTGAGT | CGAATATTTGATGCCCAAATCATAG |
| SERPINA1C | NM_009245 | GGAACCCAAGGAAAGATAGCTGA | GTCTCATCCATGGTCAGCACAG |
| ITIH1 | NM_008406 | TGGAAGTGACTCCTCAGAACATTAC | GGTCAGAGCCTGGGTGGA |
| ITIH3 | NM_008407 | GGTGACCAAGCCTGAGGACA | CGGTGACTGTCCACCACGTA |
| KLKB1 | NM_008455 | ACAATTTATACCAACTGTTGGGT | TTCACCCCAGCTGGTGAT |
| MUP19 | NM_001135127 | TCCGAATTATCTATGGTTGCTGAC | TGGCATTGGATAGGTCAATGAT |
| C8B | NM_133882 | GGCTTTGTGTGTGCACAGACA | CAGTCATTGTCCCCATTGCA |
| FNDC5 | NM_027402 | AGAAGGATGTGCGGATGCT | TCATACTGGCGGCAGAAGA |
| IL15 | NM_008357 | GCTGGCATTCATGTCTTCATTTT | GTGTTGATGAACATTTGGACAAT |
| DHRS7C | NM_001013013 | AAGGCTGGTGCTGTGTGGAAA | GCTCGGAGGCAGTCAAAGAA |
| GPC1 | NM_016696 | TGGACTGCCTGGGCAA | CTGGGAGGCTGATCCAGAA |
| SPINK3 | NM_009258 | ACCACTCCTCAGTTTCTGAAGAGAA | AGTCCCACACACAGGATCATAAA |
| SECTM1B | NM_026907 | TAAGGATTCATGGCAGCTTCATATT | TGCCGTCCTCAGTGCAAA |
| WFDC15B | NM_138685 | TCGCAGTGACCATTCTGCTT | ATCACATGTGTTTTCTCCGGTTT |
| SERPINA1F | NM_026687 | CACCAGACTGAAGAGCCATCAT | ACAAGTAATGCATGGCCATGTTA |
| C1QTNF3 | NM_001204134 | ATGGCCCCAAAGGAGAGAA | GTGCATAAGGTACACATACACTTCCTC |
| CGREF1 | NM_001160149 | TGCTCGAGACCCAGGACCT | TCTCCCTGAGCTGCACAGAAA |
| DEFB29 | NM_001001444 | ACTGTGGTGGTCGTGCTGAT | TCTCATATTTCTGGCAGGCATTT |
| SERPINA3N | NM_009252 | AACACCCTGGAAGAGATTCTAGAAG | TGAACGTGTCAAGAGGGTCAAA |
| LRG1 | NM_029796 | GCCACCTTCCTGGGGTCT | GTCACAGATCCAGGGGTTG |
| CYC | M60456 | GTTCCATCGTGTCATCAAGGACT | GCCGGAGTCGACAATGATG |
