# Supplementary material for: Identification of CTLA2A, DEFB29, WFDC15B, SERPINA1F and MUP19 as Novel Tissue-Specific Secretory Factors in Mouse
Source: PLoS One. 2015 May 6;10(5):e0124962. doi: 10.1371/journal.pone.0124962 (PMC4422522; doi:10.1371/journal.pone.0124962)
Supplement: S2 Table — (DOCX) [file pone.0124962.s002.docx]

**Table S2.** Primer sequences for PCR amplification

| Gene name | Primer sequences (5’-3’) |
| --- | --- |
| CTLA2A | F1: TGGACAACAAAATGATGGTTTCTATCT |
|  | F2: CTGCTTGGGAATGATGTCAGCT |
|  | R: CTCAAGCGTAGTCTGGGACGTCGTATGGGTACTCTGGCTGAGCCCTTCCA |
| DEFB29 | F: CCTCAGCATGCCAGTCACAA |
|  | R: CTCAAGCGTAGTCTGGGACGTCGTATGGGTAGAAGCTGGTAATCTTCCTAGGATATTTG |
| MUP19 | F: TCCCTACCAAAATGAAGATGCTGTT |
|  | R: CTCAAGCGTAGTCTGGGACGTCGTATGGGTATTCTCGGGCCTGGAGGC |
| SERPINA1F | F: CAAGATCATTTCCAGGATGACAACA |
|  | R: CTCAAGCGTAGTCTGGGACGTCGTATGGGTAGTTCTGGGGATTTACCACTCTGC |
| WFDC15B | F: TTCTTGGGAGATGAAGCTGCTT |
|  | R: CTCAAGCGTAGTCTGGGACGTCGTATGGGTATTCTGGGCTCTCCCATGGAT |
